# *De Novo* Assembly of the Genome of the Sea Urchin *Paracentrotus lividus* (Lamarck 1816)

**DOI:** 10.3390/ijms25031685

**Published:** 2024-01-30

**Authors:** Maria Costantini, Roberta Esposito, Nadia Ruocco, Davide Caramiello, Angela Cordella, Giovanna Maria Ventola, Valerio Zupo

**Affiliations:** 1 Stazione Zoologica Anton Dohrn, Department of Ecosustainable Marine Biotechnology, Via Ammiraglio Ferdinando Acton n. 55, 80133 Napoli, Italy; roberta.esposito@szn.it; 2 Stazione Zoologica Anton Dohrn, Department of Ecosustainable Marine Biotechnology, Calabria Marine Centre, C.da Torre Spaccata, 87071 Amendolara, Italy; nadia.ruocco@szn.it; 3 Stazione Zoologica Anton Dohrn, Department of Marine Animal Conservation and Public Engagement, Villa Comunale, 1, 80121 Naples, Italy; davide.caramiello@szn.it; 4 Genomix4Life S.r.l., Baronissi, 84081 Salerno, Italy; angela.cordella@genomix4life.com (A.C.); giovanna.ventola@genomix4life.com (G.M.V.); 5 Genome Research Center for Health-CRGS, Baronissi, 84081 Salerno, Italy; 6 Stazione Zoologica Anton Dohrn, Department of Ecosustainable Marine Biotechnology, Ischia Marine Centre, 80121 Naples, Italy

**Keywords:** genes, genomic resources, sea urchin

## Abstract

The Mediterranean purple sea urchin *Paracentrotus lividus* (Lamarck 1816) is a remarkable model system for molecular, evolutionary and cell biology studies, particularly in the field of developmental biology. We sequenced the genome, performed a *de novo* assembly, and analysed the assembly content. The genome of *P. lividus* was sequenced using Illumina NextSeq 500 System (Illumina) in a 2 × 150 paired-end format. More than 30,000 open reading frames (ORFs), (more than 8000 are unique), were identified and analysed to provide molecular tools accessible for the scientific community. In particular, several genes involved in complex innate immune responses, oxidative metabolism, signal transduction, and kinome, as well as genes regulating the membrane receptors, were identified in the *P. lividus* genome. In this way, the employment of the Mediterranean sea urchin for investigations and comparative analyses was empowered, leading to the explanation of cis-regulatory networks and their evolution in a key developmental model occupying an important evolutionary position with respect to vertebrates and humans.

## 1. Introduction

**Figure 1:**
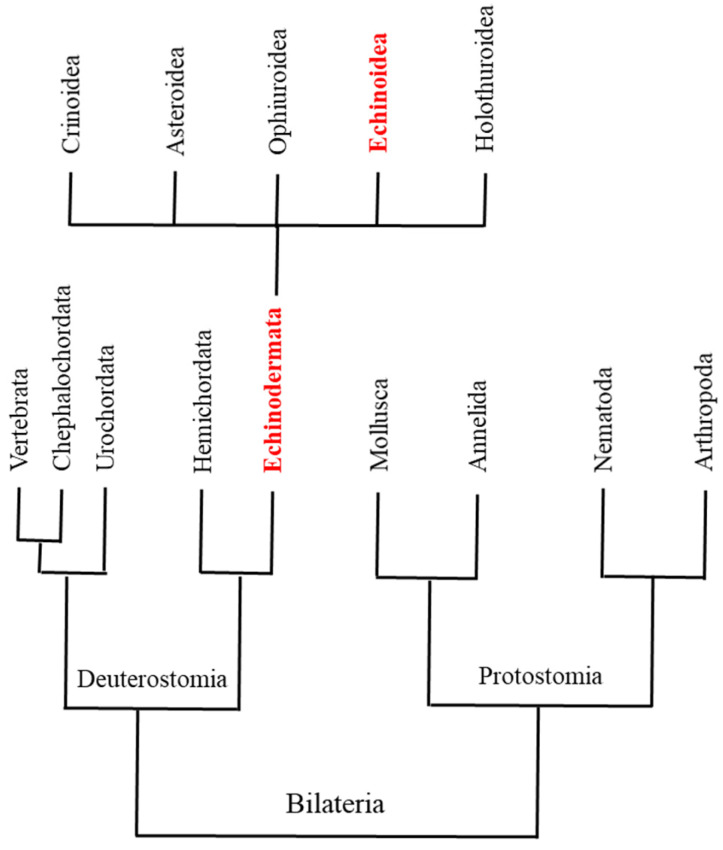
A simplified phylogenetic tree for Deuterostomia and Protostomia (according to information in Sodergren et al. [7]). Deuterostomia Echinodermata are highlighted in red font. The five classes of Echinodermata are also reported; Echinoidea class is highlighted in red.

The introduction of the Sanger method of polymerase-based sequencing revolutionized molecular/genomic studies in the early 1970s, permitting the definition of individual genomes and their regulation [1,2,3]. Next-generation sequencing (NGS) platforms increased the power of massive DNA sequencing to digitally interrogate genomes on a revolutionary scale, allowing functional genomic studies of gene expression profiling, genome annotation, and epigenetic modifications of histones or DNA methylations [4]. Availability of these omics approaches directly improved ecological genomics and/or molecular ecology studies [5]. Understanding the molecular responses of organisms to environmental stress is critical to current research on the environmental effects of global warming, ocean acidification, and increasing pollution. In this view, sea urchins are ideal models for monitoring marine environmental hazards [6], and as deuterostomes [7,8,9,10,11,12] (Figure 1), they are a perfectly positioned outgroup to the chordates [13].

Among sea urchins, *Strongylocentrotus purpuratus* and *Paracentrotus lividus* are well-established model organisms for developmental and ecotoxicological studies; the genome of the former was sequenced in 2006 [7], yielding important insights into the evolution of deuterostomes. *Paracentrotus lividus* (Parechinidae) [8], in particular, has wide geographical distribution, inhabiting shallow marine environments in the Mediterranean Sea and the eastern Atlantic Ocean [12], and is a keystone herbivore often controlling the standing crop of algal turfs and seagrass meadows [9]. In some areas, sea urchins transform macrophyte communities into barren areas, reducing biodiversity and altering ecosystem function [7,8,9,10,12,13]. Gonads of *Paracentrotus lividus* are considered a food delicacy [10,11] and it is thus intensively exploited in many Mediterranean areas. Moreover, *Paracentrotus lividus* is a well-established model for evo-devo and toxicology investigations because of the peculiar transparency of its embryos, which follow well-defined temporal patterns of development. In spite of the importance of *Paracentrotus lividus* for the ecology of coastal areas and as a model for scientific research, it was only very recently that a chromosome-scale genome assembly for this sea urchin was published [14], also reporting extensive gene expression and the epigenetic profiles of embryonic development. In addition, several SRA experiments and RNA sequences are already available on the website of the National Centre for Biotechnology Information (NCBI), which represents a relevant genomic resource. 

We present here a draft of the genome sequence of the sea urchin *Paracentrotus lividus*, with a *de novo* assembly and analysis of its content. Our findings provide a remarkable resource to elucidate the genetic mechanisms underlying the adaptation and resilience of this key grazer and, consequently, they will be of great significance for theoretical and applied research. The genome presented here will provide a paradigm for studying novel features in model animals, such as molecular pathways underlying important physiological processes, and will represent an additional resource for the conservation and management of this widely distributed marine resource.

## 2. Results and Discussion

### 2.1. Sequencing and Annotation of Paracentrotus Lividus Genome

An important node in the deuterostome phylogeny is represented by the position of Echinodermata as an early branch, implying information on vertebrate biology [13,15,16]. Among sea urchins, the family Strongylocentrotidae represents the best-studied group [17], including the species for which the genome is available, *Strongylocentrotus purpuratus* [7]. Strongylocentrotidae contain several species of marine echinoids, including four genera: *Strongylocentrotus*, *Mesocentrotus*, *Hemicentrotus* and *Pseudocentrotus* [18,19,20] (Table 1).

**Figure 2:**
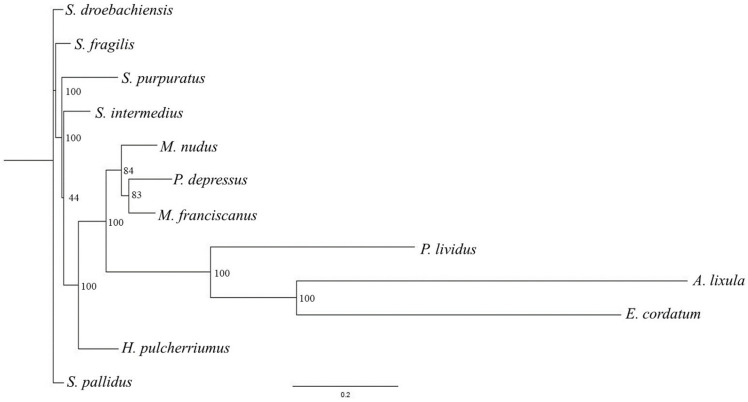
Phylogenetic tree (produced using RAxML, ML + rapid bootstrap) based on complete mitochondrial genomes available for 12 sea urchin species downloaded from GenBank: *Allocentrotus fragilis* (KC89820); *Arbacia lixula* (NC_001770); *Echinocardium cordatum* (FN562581); *Hemicentrotus pulcherrimus* (NC_023771); *Mesocentrotus franciscanus* (NC_024177); *Mesocentrotus nudus* (NC_020771); *Pseudocentrotus depressus* (NC_023773); *Strongylocentrotus droebachensis* (NC_009940); *Strongylocentrotus intermedius* (NC_023772); *Strongylocentrotus pallidus* (NC_009941); *Strongylocentrotus purpuratus* (NC_001453); and *Paracentrotus lividus* (NC_001572). The values are reported as percentage of node label. Data on nucleotides are reported in Table S1.

The phylogenetic tree reported in Figure 2 was based on complete mitochondrial genomes available for 12 sea urchin species, and *Paracentrotus lividus* was selected to root the mitochondrial trees because it is strongly supported as an appropriate outgroup for Strongylocentrotidae, with an estimate of 35–50 Myr used as a reference time point for the split between the Strongylocentrotid species and Parechinids [21].

**Figure 3:**
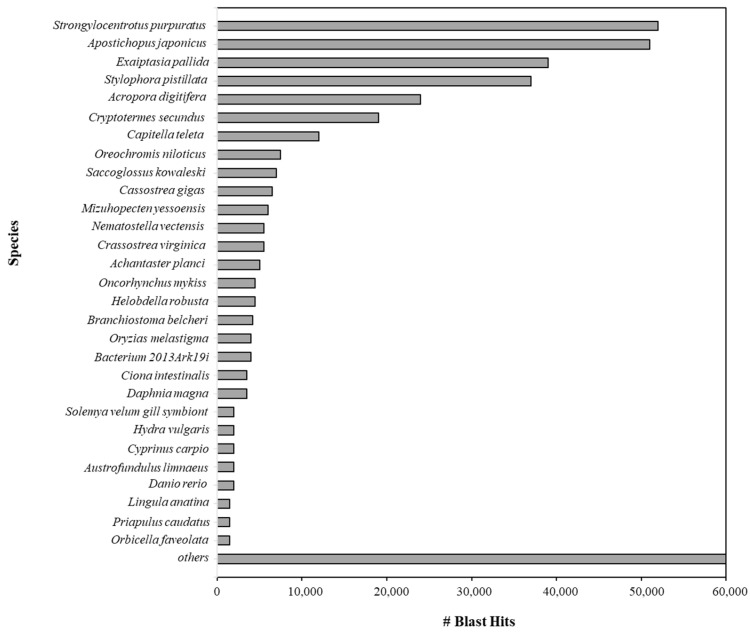
BLAST top hit species distribution (reported as number) of matches with known sequences aligned during the BLAST step using the NCBI database. In total, 3142 species were obtained with at least one BLAST hit, but in the histogram only the top thirty hits were reported; the remaining species are listed in “Others”.

BLAST top hit species distribution of matches for all the scaffolds with known sequences indicated that the majority of *P. lividus* scaffolds show the highest homology with *S. purpuratus* (BLAST Hits = 52,000) (Figure 3).

The most-represented species included *Apostichopus japonicus* (sea cucumber, BLAST hits: 51,000); *Exaiptasia pallida* (sea anemone, BLAST: hits 39,000); and *Stylophora pistillata* (coral, BLAST hits: 37,000). All alignments were carried out by setting the E-value thresholds as ≤1 × 10^−5^. By using ABySS (version 2.0), 252,999 contigs and 252,952 scaffolds were obtained (Table 2). ABySS represents a resource-efficient assembly of large genomes using a Bloom filter [22].

The maximum scaffold length for the genome was 6805 nucleotides with an N50 of 792 (min length 500 bp). The total length of the scaffolds, considering contigs ≥ 500 bp, was 1,486,080 nucleotides and the GC content corresponded to 31.9%. Using Geneious, 337,545 ORF sequences were obtained, belonging to 140,726 unique scaffold sequences. The annotation of ORF sequences (8508 were unique) was performed using Blast2GO, obtaining 33,529 ORF. Of these sequences, 13,523 and 21,380 were identified with InterPro and GO IDs, respectively, as shown in Table S2.

**Figure 4:**
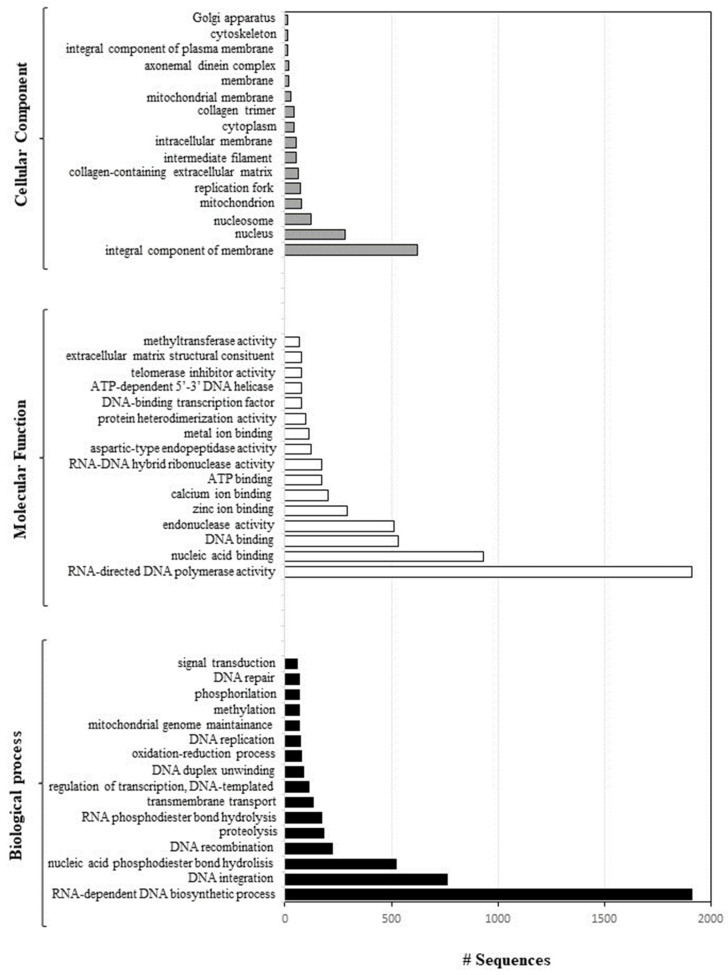
Direct GO count distribution, showing Molecular Function (a chart for the Molecular Function GO category, which shows the most frequent GO terms within a data-set without taking into account the GO hierarchy: white bars), Biological Process (same as above but for Biological Process: black bars), and Cellular Component (same as above but for Cellular Component: grey bars).

A total of 48 GO terms were enriched, including 16 in “Biological Process” followed by 16 in “Molecular Function” and 16 in “Cellular Component” (*p* < 0.05) (Figure 4).

**Figure 5:**
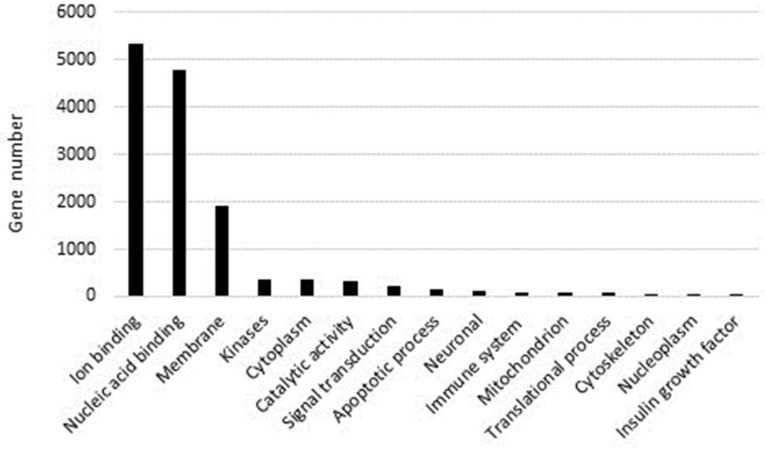
Analysis of *P. lividus* genes reporting the gene number involved in different cellular processes.

Over-represented GO categories included RNA-dependent DNA biosynthetic process; DNA integration; nucleic acid phosphodiester bond hydrolysis; DNA recombination; RNA-directed DNA polymerase activity; nucleic acid binding; DNA binding endonuclease activity; zinc ion binding; calcium ion binding; and DNA binding (see also Figure 5).

Moreover, these genes are an integral component of the cell membrane and were mainly localised in the nucleus, nucleosome, mitochondrion, and replication fork. 

The draft genome of *P. lividus* reported here provided additional information to those reported in Marlétaz et al. [14], allowing for a comprehensive survey of the main gene pathways available for further developmental investigations. Evidently, the peculiar phylogenetic position of echinoderms offers the possibility to perform comparisons between protostomes and deuterostomes, and between invertebrate and vertebrate deuterostomes. Many aspects of development and cell–cell interactions will provide new perspectives on those genes that evolved to control important developmental processes. Sea urchins are evolutionarily closer to not only other deuterostomes, including vertebrates, but also to protostomes, such as *Drosophila* and *Caenorhabditis elegans*. From this perspective, they may provide a clearer view into the evolution of vertebrates, including their developmental signalling and evolution. Evidently, sea urchin embryos are a key evolutionary link to vertebrate development, although the extent of molecular commonality has only now become measurable with the genomic sequence data.

### 2.2. Key Findings and Genes

#### 2.2.1. Complex Innate Immune Responses 

Among the classes of innate receptors, toll-like receptor (TLR) genes from 48 proteins, TLR-1, TLR-2, TLR-3, TLR-4, were identified (see Table 3). TLRs represent an important part of innate immunity, playing key roles in the defence against pathogen invasion [23]. 

Cell surface TLRs include TLR1; TLR2; TLR4; TLR5; TLR6; and TLR10, whereas intracellular TLRs include TLR3; TLR7; TLR8; TLR9; TLR11; TLR12; and TLR13, which are localised in the endosome [24]. Even if TLRs were first described as important recognition receptors in mice and humans, they were also extensively studied in several animal species, including invertebrates such as the sea urchin, where a great expansion of TLR genes occurred. Gene expression analysis reported TLR receptors specifically expressed at the two-cell cleavage stage [14].

Despite the apparent simplicity of their body organization, echinoderms exhibit an immune system able to perform complex innate immune responses, a phenomenon which is, to date, quite far from being completely understood [25]. In fact, the *S. purpuratus* genome represents a window on the functions of the innate immune system complexity and sensing capacity, revealing a close genetic relationship between sea urchins and humans and thus reinforcing the relevance of these model organisms. In addition, echinoderms are advanced invertebrates, representing a bridge with primitive chordates, because they possess numerous receptors and effectors used to obtain a rapid immune response. After an infection, their cellular immune response triggers a network formed by membrane and endosomal receptors, which in turn triggers an immune response by stimulating consecutive intracellular events [26,27].

The genome of this sea urchin holds a vast set of at least 222 TLR genes, accompanied by a moderate expansion of downstream adaptors, different from that of chordates [28,29,30]. The abundance of TLRs in sea urchins suggests that this class of receptors plays an important role in the innate immune defence, possibly the case in lower animals as well. The vast majority of sea urchin TLR genes are more similar to each other than to those of other animals, suggesting a gene expansion specific to the sea urchin lineage [28]. The recognition of non-self molecules by specific membrane receptors triggers the immune response, stimulating consecutive intracellular events [30]. An E3 ubiquitin-protein ligase pellino homolog 1 gene was identified, as it has been for *S. purpuratus* (LOC577851). This gene, located in the cytosol, enables ubiquitin protein ligase activity and is involved in several processes, including the negative regulation of the necroptotic process; it also participates in protein poly-ubiquitination. 

#### 2.2.2. Molecular Switches in Signal Transduction

Our data revealed for the first time the presence in the *Paracentrotus lividus* genome of four genes belonging to the small guanosine triphosphatase (GTPases) families: Ras, Rab, Ral, ARF and Rho. Comparing these data with those reported in the case of the genome of *S. purpuratus*, four families of RAS GTPases are in common with humans: Ras, Rho, Rab, and ARF. The genes of this family are usually expressed during embryogenesis [18,31]. They represent a large family of hydrolase enzymes able to bind to the nucleotide guanosine triphosphate (GTP), and hydrolyse it to guanosine diphosphate (GDP), which in turn functions as a molecular switch in signal transduction, nuclear import and export, lipid metabolism, and vesicle docking [7]. Vertebrate GTPase families were expanded after their divergence from echinoderms, thanks to whole genome duplications [19,20]. This is different from the sea urchin genome, which did not undergo a whole-genome duplication. In the case of four Ras GTPase families, (Ras, Rho, Rab, and Arf), a local gene duplication occurred, resulting in a comparable number of monomeric GTPases in the genomes of humans and sea urchins, and signalling complexity mediated by GTPases. 

Another gene involved in signal transduction processes is 1-phosphatidylinositol 4,5-bisphosphate phosphodiesterase beta-4 (PLCB4), also identified in the genome of *P. lividus*. Phosphatidylinositol-specific phospholipase C enzymes mediate the production of the second messenger molecules diacylglycerol (DAG) and inositol 1,4,5-trisphosphate (IP3); in humans this form has a role in retina signal transduction [32]. The protein encoded by this gene is a phosphodiesterase, which catalyses the hydrolysis of phosphatidylinositol 4,5-bisphosphate to the second messengers, inositol 1,4,5-trisphosphate (IP3) and diacylglycerol. The encoded protein is activated by G proteins and is involved in the signal transduction pathway of the type-2 taste receptor. In addition, the nuclear factor kappa B (which is also identified in *P. lividus* genome) can regulate the transcription of this gene, whose protein product can also act as an important regulator of platelet responses.

Another interesting gene here found (orthologous, also found in *S. purputarus*), is the allatostatin-A receptor-like gene, which belongs to the type A allatostatins (AST-As), a family of insect peptides with a conserved C-terminal FGL-amide motif [33]. The insect allatostatin-A receptors (AST-ARs), considered orthologues of galanin receptors (GALR) in vertebrates [34], are activated by AST-A peptides. Vertebrate GALR receptors have a close relationship, from an evolutionary point of view, with kisspeptin receptors (KISSR), and are in turn activated by galanin (GAL) and spexin (SPX) peptides, which are unrelated to insect AST-As. 

In contrast, the ankyrin (ANK) repeat protein family is largely distributed across plants and has been found to participate in multiple processes such as plant growth and development, hormone response, and response to biotic and abiotic stresses [35]. 

Finally, the calcium-independent protein kinase C (PKC) has a key role in signal transduction mechanisms. In particular, this gene is involved in the initiation and maintenance of motility in the spermatozoa of the sea urchin *Lytechinus pictus*. White et al. [32] demonstrated the existence of a correlation between motility and the level of phospho-PKC substrates, so PKC activation and phosphorylation of its target proteins represent a significant requirement for the maintenance of motility in the spermatozoa of intact sea urchins. In *S. purpuratus*, the levels of PKC substrates contribute to the production of immotile and motile spermatozoa, and non-competitive PKC inhibitors are involved in diminishing the circular velocity of spermatozoa [36]. 

#### 2.2.3. Genes Regulating the Membrane Receptors 

The alpha-like subunit of the acetylcholine receptor (AChR) binds acetylcholine, and just after is subject to an extensive change in its conformation, thereby affecting all subunits and the opening of an ion-conducting channel present in the plasma membrane. ATPases comprise a superfamily of proteins involved in several cellular processes essential for physiology, (control of proteins; homeostasis; DNA replication; recombination; chromatin re-modelling; ribosomal RNA processing; molecular targeting; organelle biogenesis; and membrane fusion); they are often associated with diverse cellular activities (AAA+) [37,38,39,40]. In fact, the members of this superfamily are defined by the presence of the AAA+ domain, containing the canonical Walker A and B motifs necessary for ATP binding and its hydrolysis [37]. 

The receptor for egg jelly precursor is an integral component of the membrane, with an important role in calcium channel activities and ion binding. Three of these receptors, suREJ1, suREJ2, and suREJ3, were previously described in *S. purpuratus* testis [41]. In particular, suREJ1 is composed of one transmembrane segment, able to bind to the fucose sulphate polymer of egg jelly, thus inducing the sperm acrosome reaction. On the other hand, suREJ3 consists of 11 putative transmembrane segments localised in the plasma membrane over the acrosomal vesicle. In contrast, suREJ2 is an intracellular plasma membrane protein with no extracellular projection from the plasma membrane, and two transmembrane segments; it is present in the entire spermatozoa plasma membrane, mainly concentrated over the spermatozoan mitochondrion. REJ is a common module present in all three sea urchin sperm REJ proteins, and is shared by the human autosomal dominant polycystic kidney disease protein, polycystin-1, and PKDREJ—a testis-specific protein found in mammals [42]. Ankirin is another integral protein component of the membrane, involved in protein heterodimerization. The ankyrin (ANK) repeat domain was identified for the first time in some yeast cell-cycle regulators, and in the *Drosophila melanogaster* signalling protein Notch3 [43,44]. It is considered the most common conserved protein domain, being distributed in organisms ranging from viruses to humans [35,45]. Yaguchi et al. [46] isolated the gene ankAT-1, the Ankyrin-containing gene specific for apical tuft, the expression of which is normally found in the animal pole region of the very early blastula stages of sea urchin embryos. This gene is involved in the regulation of the length of apical tuft cilia, mediating apical tuft formation in the sea urchin embryo, as demonstrated by experimental knock-down of this gene, resulting in much shorter embryos and with decreased motility with respect to the motile cilia in other ectodermal cells^46^. Although the specific functions of the ANK domain proteins are still not known, they were identified in several proteins with diverse functions, highlighting their roles as mediators of protein–protein interactions and acting as molecular chaperones [42,47,48,49].

Lamin B is a structural constituent of the extracellular matrix, with a key role in calcium ion binding and cell-matrix adhesion. In the sea urchin *L. pictus*, it is imported into the nucleus from a soluble pool at a later stage of pronuclear formation; the resulting incorporation is necessary for pronuclear swelling and growth of the nuclear envelope [50].

The genome of *Paracentrotus lividus* possesses a putative precursor of fibrillin-2, a matrix protein involved in protein kinase activity, ATP binding, and protein phosphorylation. In the *S. purpuratus* genome, two genes encoding fibrillin homologs were identified, suggesting an expansion of this family in deuterostomes, clustering with vertebrate, honeybee and ascidian fibrillins [51]. Fibrillin A is expressed during cleavage and by primary mesenchyme cells, with a role in the fibrillar components of the blastocoel extracellular matrix [52]. There are three fibrillin genes in mammals (FBN-1, FBN-2, and FBN-3), and only one each in *C. elegans* (fbn-1) and *Drosophila* (CG31999). Mutations in human fibrillins are responsible for Marfan syndrome and the related disease, contractural arachnodactyly. 

Rhodopsin is a light-sensitive receptor protein belonging to G-protein-coupled receptors (GPCRs), involved in photo-transduction and contributing to the majority of sensory receptors in vertebrates. In the sequenced sea urchin genome, they represent the largest GPCR family with 979 members constituting more than 3% of all predicted genes [53]. Moreover, four greatly expanded subfamilies of rhodopsin-type GPCRs were identified, which rapidly expand the lineages of GPCRs (surreal-GPCRs). This group is mostly expressed in different classes of pedicellariae and in the tube feet of adult sea urchins, harbouring sensory neurons involved in the reaction to chemical stimuli in echinoderms. In addition, these structures also express different opsins, indicating that sea urchins possess a complex system for sensing their environment. These genes may have arisen by rapid duplication in the echinoid lineage, acting as chemosensory receptors. 

#### 2.2.4. Nervous System and Neuronal Genes

The nervous system of echinoderms is dispersed both in larvae and adults, and thus differs from both vertebrates and hemichordates, but it is not a simple nerve net. In fact, vertebrates do not have a dispersed nervous system, and hemichordates have nerve nets [54]. Adult sea urchins have thousands of appendages with sensory neurons, ganglia, and motor neurons arranged in local reflex arcs. These peripheral appendages are connected to each other and to radial nerves for overall control and coordination. 

The genome of *Paracentrotus lividus* showed the presence of a calcineurin gene. Since the 1970s, a calmodulin-binding protein was found only in the brain of sea urchins [55,56,57]. This protein, named calcineurin, is localised in neurons and is associated with post synapsis and dendrite microtubules [58]. Stewart et al. [59] found that protein phosphatase 2B, involved in glycogen metabolism in skeletal muscle, is similar to calcineurin, which in turn has similar activity.

In *S. purpuratus* there is a single predicted gene for a neurexin (a synaptic adhesion component [51]), also found in the genome of *Paracentrotus lividus*. There are several predictions for neuroligins, the postsynaptic receptors for neurexins, also known from other invertebrates, where β-neurexin binds neuroligin and is clustered to recruit presynaptic components [60].

In contrast, neurocan is a member of the lectican/chondroitin sulphate proteoglycan protein families; it consists of neurocan core protein (identified in the *Paracentrotus lividus* genome) and chondroitin sulphate. It is involved in the modulation of cell adhesion and migration. The neuroendocrine convertase 1 gene isolated in *Paracentrotus lividus* has some orthologs in *S. purpuratus*, such as furin and subtilisin, which may process TGF-β precursors [61]. The neurogenic locus Notch protein has a neural ortholog in the sea urchin *Lytechinus variegatus*. The Notch intercellular signalling pathway mediates the specification of numerous cell fates in both invertebrate and vertebrate development [62]. The navigator 3-like neuron belongs to the neuron navigator family and is expressed predominantly in the nervous system. Genes related to neurotransmitter receptors were also found, such as the neuronal acetylcholine receptor subunit alpha-5-like gene. These genes also include beta-adrenergic receptor kinase 2 and are confirmed to be localised to a subset of ectoderm, consistent with a neural population [63].

#### 2.2.5. The Kinome of *P. lividus* Resembles That of Drosophila and Human

Several kinases were identified in the genome of *Paracentrotus lividus*: 52 kDa repressor of the inhibitor of the kinase-like protein; adenosine kinase; A-kinase anchor protein 17A; bifunctional UDP-N-acetylglucosamine 2-epimerase/N-acetylmannosamine kinase isoform X1; cell division cycle 7-related protein kinase; 2-like cyclin-dependent kinase; dolichol kinase; dual specificity mitogen-activated protein kinase 7 isoform X2; inositol hexakisphosphate and diphosphoinositol-pentakisphosphate kinase 1 isoform X1; L-fucose kinase; MAP kinase-activating death domain protein isoform X1; MAPK/MAK/MRK overlapping kinase; maternal embryonic leucine zipper kinase isoform X2; membrane-associated guanylate kinase; WW and PDZ domain-containing protein 2-like; erbB-4-like receptor tyrosine-protein kinase; receptor-like protein kinase Feronia; serine/threonine-protein kinase PAK 2; Tie-1-like tyrosine-protein kinase receptor; and wall-associated receptor kinase and wee1-like protein kinase 1-A. All these genes define the kinome of *Paracentrotus lividus*, representing the complete set of protein kinases encoded in the genome [64]. The genomic sequence of *S. purpuratus* and the predicted gene models were used to identify the predicted protein kinases in this genome, according to both function and kinase domain taxonomy. The results showed that the sea urchin kinome: i, consists of 353 protein kinases, and ii, is closer to the *Drosophila* kinome (239) than the human kinome (518), according to the total kinase number. However, it has been established that the diversity of sea urchin kinases is surprisingly similar to those of humans. In fact, the urchin kinome misses only 4 of 186 human subfamilies, while *Drosophila* lacks 24, thus combining the simplicity of a non-duplicated genome with the diversity of function and signalling, which was previously considered to be very specific to vertebrates [64]. More than half of the sea urchin kinases are involved in signal transduction, and approximately 88% of the signalling kinases are expressed in the developing embryo. An example is the dolichol kinase, for which Rossignol et al. [65] reported that both *de novo* synthesis of dolichol and its phosphorylation may play an important role in the observed increase of glycoprotein synthesis in early embryonic development, prior to gastrulation. Protein kinases play crucial roles in the regulation of signalling pathways, in coordination with protein phosphatases, in the genome of *S. purpuratus* [66]. In fact, high expression of kinases was detected in endomesoderm formation. 

#### 2.2.6. Homologies with Human Oxidative Metabolism

About 30 CYP-related genes were identified in the *Paracentrotus lividus* genome, mostly belonging to CYP 1, 2, and 3. Several CYP 2 genes (28) were found, while nine CYP1-like genes and only three CYP 3 genes (CYP3-like) were detected. The genome of *P. lividus* contains homologs of proteins involved in metabolism (CYP 27), and three other families: the CYP 6, CYP 20, and CYP 120 genes. These enzymes, belonging to the CYP1, CYP2, CYP3, and CYP4 families, play key roles in the oxidative biotransformation of chemicals to more hydrophilic products. The sea urchin *S. purpuratus* contains 120 CYP genes, of which 80% of the total are related to CYP gene families 1 to 4, suggesting a selective pressure to expand functionality in these gene families.

Eleven CYP1-like genes are present in the genome of *Paracentrotus lividus*, which represent more than twice the number of these genes among chordates. A greater amount of CYP2-like and CYP3-like genes are also observed in comparison to other deuterostomes. In addition to the CYPs in families 1 to 4, the sea urchin genome contains homologs of proteins involved in developmental patterning (CYP26), cholesterol synthesis (CYP51), and metabolism (CYP27 and CYP46). Homologs of some CYPs with endogenous functions in vertebrates are not found. These CYP genes, in concert with additional expanded defensive gene families, represent a large diversification of defence gene families by the sea urchin relative to mammals. 

## 3. Materials and Methods

### 3.1. Ethics Statement

*Paracentrotus lividus* (Lamarck) were collected from a site in the Bay of Naples that is not privately owned or protected in any way, according to Italian legislation (DPR 1639/68, 19 September 1980, confirmed on 10 January 2000). Field studies did not include endangered or protected species. All experimental procedures on animals were in compliance with the guidelines of the European Union (Directive 609/86).

### 3.2. Sample Collection and DNA Extraction 

Adult sea urchins were collected during the breeding season during scuba-diving in the Gulf of Naples, transported in a thermic box to the laboratory within 1 h after collection and maintained in tanks with circulating sea water until testing [67]. Sea urchins were injected with 2 M KCl through the peribuccal membrane to obtain the emission of gametes. Concentrated spermatozoans were collected and immediately used for DNA extraction. Genomic DNA was extracted from single male sperm using 1× TEN buffer (50 mM Tris pH 7.6, 10 mM EDTA, 100 mM NaCl) plus 1% sodium dodecyl sulfate. Digestion with proteinase K (100 mg/mL) was performed at 55 °C overnight, and contaminating RNA was degraded by treating RNase (10 mg/mL) at 37 °C for one hour. Extraction with phenol–chloroform–isoamyl alcohol (25:24:1) was then followed by precipitation via the addition of 3M sodium acetate/95% ethanol. The amount of total DNA extracted was estimated by the absorbance at 260 nm. 

### 3.3. De Novo Genome Assembly

The pipeline followed for the genome assembly and annotation was as follows:

1. Genome sequencing: the next generation sequencing experiment and bioinformatics analysis were performed using Genomix4life S.R.L. (Baronissi, Salerno, Italy). DNA concentration was assayed with a ND-1000 spectrophotometer (NanoDrop, ND-1000 UV-Vis Spectrophotometer; NanoDrop Technologies, Wilmington, DE, USA), and its quality assessed with an Agilent 4200 Tapestation (Agilent Technologies, Santa Clara, CA, USA; according manufacturer instructions). An indexed library was prepared from 1 µg of purified DNA with a Truseq DNA Nano Library Prep Kit according to the manufacturer’s instructions (Illumina, San Diego, CA, USA). The library was quantified using the Tape Station 4200 (Agilent Technologies, Santa Clara, CA, USA) and a Qubit fluorometer (Invitrogen Co., Carlsbad, CA, USA), and diluted with a final concentration of 2 nM. The sample was subject to cluster generation and sequencing using an Illumina NextSeq 500 System (Illumina) in a 2 × 150 paired-end format, according NextSeq 500 System Documentation.
2. Sequencing outputs, quality control and cleaning: the most common metric was used to assess the accuracy of a sequencing platform (base calling accuracy, measured by the Phred quality score (Q score). The first step was a quality check of the raw Illumina sequencing data to remove adapter sequences and low-quality reads, using ad hoc script. The FastQC tool (available on http://www.bioinformatics.babraham.ac.uk/projects/fastqc; 1 February 2021) was used to check the quality of raw data sequencing.
3. Genome assembly: to perform the *de novo* assembly, a KmerGenie (version 1.7044) tool was necessary to estimate the best k-mer length 66. In this case, the best k-mer predicted was 121. ABySS 2.0, an implementation of ABySS 1.0, was used to perform the *de novo* assembly on fastq files. The bloom filter of ABySS 2.0 was applied to avoid duplicate sequences.
4. Genome assembly stats and validation: the integrity assembly was also evaluated, using several statistical tools, such as QUAST, Abyss, BBMAP, and BUSCO (Table S3).
5. Genome annotation and functional analysis: Geneious software 69 was used to identify all the ORF sequences, and Blast2GO was applied to perform a blast alignment of all ORF sequences identified and to annotate everything in the Gene Ontology database.

The assembly has been deposited in the SRA database (submission ID: SUB6921168; BioProject ID: PRJNA604684; BioSample: Processed Successfully loaded SAMN13978365: PARLIV_1.0; TaxID: 7656). This whole-genome shotgun project has been deposited at DDBJ/ENA/GenBank under the accession JAWLRT000000000. The version described in this paper is version JAWLRT010000000.

### 3.4. Phylogenetic Tree

The phylogenetic tree was based on complete nucleotide mitochondrial genomes available for 12 sea urchin species: *Allocentrotus fragilis* (KC89820); *Arbacia lixula* (NC_001770); *Echinocardium cordatum* (FN562581); *Hemicentrotus pulcherrimus* (NC_023771); *Mesocentrotus franciscanus* (NC_024177); *Mesocentrotus nudus* (NC_020771); *Pseudocentrotus depressus* (NC_023773); *Strongylocentrotus droebachensis* (NC_009940); *Strongylocentrotus intermedius* (NC_023772), *Strongylocentrotus pallidus* (NC_009941); *Strongylocentrotus purpuratus* (NC_001453); and *Paracentrotus lividus* (NC_001572). The sequences were aligned with SeaView [68], a software permitting the performance of a complete phylogenetic analysis of a set of homologous DNA or protein sequences, from network-based sequence extraction from public databases to tree building and display, using up-to-date alignment and a maximum-likelihood tree-building algorithm [69]. The phylogenetic tree was produced using RAxML [69] and figures with FigTree V.1.4.3 [70]. In detail, a set with the function “G blocks” was created, permitting smaller final blocks, gap positions within the final blocks, and less strict flanking positions. It reports bootstrap resampling for branch-support estimation with a substitution matrix (GTR) and substitution rates (Gamma), offering nucleotide sequence alignments that were evolved with non-stationary (NS) and non-reversible (NR) substitution models.

## 4. Conclusions

This *de novo* assembly greatly expands on the previous analysis of the *Paracentrotus lividus* sea urchin genome reported very recently by Marlétaz et al. [14], highlighting genomic and regulatory evolution in deuterostomes. In fact, our data added new information on several classes of genes which have not been previously identified. Several genes involved in complex innate immune responses, oxidative metabolism, signal transduction, and kinome, as well as genes regulating the membrane receptors, were identified analysing the content of the assembly. This represents a very significant step in understanding the evolution of this key species, not only by the deuterostome phylogeny implying information on vertebrate biology, but also with respect to the vertebrates themselves, which we are planning to expand by adding long-read analysis. The analyses of *P. lividus* genes could also shed light on biological processes, and on differences/similarities among species or genera of sea urchins.

## Figures and Tables

**Table 1:** Species belonging to the four genera of Strongylocentrotidae.

Genera	Species
*Strongylocentrotus*	*S. purpuratus*
	*S. pallidus*
	*S. droebachiensis*
	*S. intermedius*
	*S. fragilis*
	*S. polyacanthus*
*Mesocentrotus*	*M. franciscanus*
	*M. nudus*
*Hemicentrotus*	*H. pulcherrimus*
*Pseudocentrotus*	*P. depressus*

**Table 2:** Statistical analysis of *P. lividus* genome size.

Parameter	Quast	ABYSS	BUSCO
Assembly	scaffolds (min. Length = 500 bp)	scaffolds (min. Length = 500 bp)	
contigs (≥0 bp)	252,952	252,952	252,952
contigs (≥1000 bp)	280	_	
contigs (≥5000 bp)	5	_	
Total_length (≥0 bp)	42,528,692	_	42,528,692
Total_length (≥1000 bp)	515,753	_	
Total_length (≥5000 bp)	28,242	_	
Total_length (≥10,000 bp)	0	_	
contigs	1757	1757	
Largest_contig	6806	6805	
Total_length	1,488,145	1,486,080	
GC(%)	34.77%	31.88%	
N50	792	791	153

**Table 3:** Summary of all genes/proteins identified in the genome of *P. lividus*.

	Genes/Proteins
**Immune response**	Toll-like receptor 1
	Toll-like receptor 2
	Toll-like receptor 3
	Toll-like receptor 4
	Toll-like receptor 5
	Toll-like receptor 6
	Toll-like receptor 7
	Toll-like receptor 8
	Toll-like receptor 9
	Toll-like receptor 10
	Toll-like receptor 11
	Toll-like receptor 12
	Toll-like receptor 13
	E3 ubiquitin-protein ligase pellino homolog 1
**Signal transduction**	Ras
	Rab
	Ral
	Arf
	Rhodopsin
	1-phosphatidylinositol 4,5-bisphosphate phosphodiesterase beta-4
	Nuclear factor kappa B
	Allatostatin-A receptor-like
	Calcium-independent protein kinase C
**Membrane receptors**	suREJ1
	suREJ2
	suREJ3
	Ankyrin-containing gene specific for Apical Tuft
	Fibrillin A
	Rhodopsin
**Neuronal genes**	Calcineurin
	Neurexin
	Neurocan
	Neuroendocrine convertase 1 gene
	Neuron navigator 3-like
	Neuronal acetylcholine receptor subunit alpha-5-like
	Beta-adrenergic receptor kinase 2
**Kinome**	Adenosine kinase
	A-kinase anchor protein 17A
	Bifunctional UDP-N-acetylglucosamine 2-epimerase/N-acetylmannosamine kinase isoform X1
	Cell division cycle 7-related protein kinase
	Cyclin-dependent kinase 2-like
	Dolichol kinase
	Dual specificity mitogen-activated protein kinase kinase 7 isoform X2
	Inositol hexakisphosphate
	Diphosphoinositol-pentakisphosphate kinase 1 isoform X1
	L-fucose kinase
	MAP kinase
	Maternal embryonic leucine zipper kinase isoform X2
	Membrane-associated guanylate kinase
	WW
	PDZ domain-containing protein 2-like
	Receptor tyrosine-protein kinase erbB-4-like
	Receptor-like protein kinase feronia, serine/threonine-protein kinase PAK 2
	Tyrosine-protein kinase receptor Tie-1-like
	Wall-associated receptor kinase and wee1-like protein kinase 1-A
**Oxidative metabolism**	CYP 1-like
	CYP 2-like
	CYP 3-like
	CYP 4-like
	CYP 6-like
	CYP 20-like
	CYP 26-like
	CYP 27-like
	CYP 46-like
	CYP 51-like
	CYP 120-like

## Data Availability

The assembly has been deposited in the SRA database (submission ID: SUB6921168; BioProject ID: PRJNA604684; BioSample: Processed; Successfully loaded SAMN13978365: PARLIV_1.0 (TaxID: 7656). This whole-genome shotgun project has been deposited at DDBJ/ENA/GenBank under the accession JAWLRT000000000. The version described in this paper is version JAWLRT010000000.

## Supplementary Materials

The following supporting information can be downloaded at: https://www.mdpi.com/article/10.3390/ijms25031685/s1.
